# Complete Genome Sequences of Two Chikungunya Viruses Isolated in the Central African Republic in the 1970s and 1980s

**DOI:** 10.1128/genomeA.00003-17

**Published:** 2017-03-02

**Authors:** Vianney Tricou, Marion Desdouits, Emmanuel Nakouné, Antoine Gessain, Mirdad Kazanji, Nicolas Berthet

**Affiliations:** a Department of Virology, Institut Pasteur de Bangui, Bangui, Central African Republic; b Institut Pasteur, Epidemiology and Physiopathology of Oncogenic Viruses, Paris, France; c Centre National de Recherche Scientifique (CNRS), UMR 3569, Paris, France

## Abstract

Some arboviruses threaten human global health with potentially explosive emergence. Analysis of whole-genome sequences of decades-old isolates might contribute to the understanding of the complex dynamics which drive their circulation and emergence. Here, we report the whole-genome sequences of two Chikungunya viruses isolated in the Central African Republic in the 1970s and 1980s.

## GENOME ANNOUNCEMENT

Chikungunya virus (CHIKV) is an arthropod-borne virus that belongs to the *Alphavirus* genus and *Togaviridae* family and causes disease in humans. CHIKV is transmitted to people by mosquitoes, mainly *Aedes albopictus* and *Aedes aegypti*. The most common symptoms of CHIKV infection are fever and joint pain but may include headache, muscle pain, joint swelling, or rash. CHIKV was only found in Africa, Asia, and India until recently, but outbreaks were also reported over the past decade in Europe, islands in the Indian and Pacific Oceans, and the Americas. There is no vaccine to prevent or medicine to treat CHIKV infection (1).

CHIKV has a ~12-kb-long single-stranded positive-sense RNA genome which contains two open reading frames (ORFs) flanked by two untranslated regions (UTRs), a 5′-cap, and a 3′-poly(A) tail. The first ORF encodes the nonstructural proteins NSP1 to NSP4. The second ORF encodes the structural proteins, including the capsid protein and the envelope proteins E1, E2, and E3 (2). Three lineages have been described: the West African, East-Central-South African (ECSA), and Asian lineages (3). A new lineage, derived from the ECSA, emerged in Kenya in 2004 and spread across the Indian Ocean (4).

Here, we report the whole-genome sequences of ArB6445 and HB84P07 viruses isolated from *Aedes opok* and human serum specimens, respectively. The *A. opok* specimen was collected in 1975 in Bozo, a forest area located ~150 km north of Bangui (the capital of the Central African Republic [CAR]), and the human serum specimen was collected from a febrile patient presenting to the Institut Pasteur in Bangui in 1983 with arthralgia and myalgia (5). These viruses were isolated and amplified by serial passages in brains of newborn mice. Brains were homogenized in Hanks’ solution and centrifuged. Supernatants were lyophilized and stored in sealed glass vials at room temperature until 2011. Viral genomic material was extracted from lyophilizates that were resuspended in phosphate-buffered saline, retrotranscribed into cDNA using SuperScript III enzyme and random hexamers (Life Technologies, Inc.), and amplified using the phi29 enzyme (6). Sequencing was performed using a HiSeq 2000 sequencer (Illumina) (7). Only regions of the reads matching the reference viral sequences were selected and used for the whole-genome sequence assembly using SPAdes version 3.0.0 (8, 9).

Overall lengths were 11,805 and 11,786 nucleotides, with average coverages of 2,743× and 404× for ArB6445 and HB84P07, respectively. The coding sequence lengths were 7,425 and 3,747 nucleotides for the first and second ORFs, respectively. The two viruses share >99% nucleic acid identity. Phylogenetic analyses showed close relatedness with other CHIKV isolated in the CAR and confirmed classification into the ECSA lineage, as previously described with partial sequences (5). Unlike the S27-African prototype strain (GenBank accession no. NC_004162), a leaky stop codon near the *nsP3* gene 3′ end is present in both sequences. The 3′-UTR (which plays essential roles in arbovirus replication, evolution, and host adaptation) of both viruses exhibits the typical duplication pattern of other ECSA lineage strains (10). Further investigations might help better understand the recent evolutionary history of CHIKV.

### Accession number(s).

The whole-genome sequences of ArB6445 and HB84P07 are available in DDBJ/EMBL/GenBank databases under accession numbers KY038946 and KY038947, respectively.
